# Bacterial Expression of Human Butyrylcholinesterase as a Tool for Nerve Agent Bioscavengers Development

**DOI:** 10.3390/molecules22111828

**Published:** 2017-10-27

**Authors:** Xavier Brazzolotto, Alexandre Igert, Virginia Guillon, Gianluca Santoni, Florian Nachon

**Affiliations:** 1 Institut de Recherche Biomédicale des Armées, Département de Toxicologie et Risques Chimiques, 1 Place Général Valérie André, 91223 Brétigny-sur-Orge, France; alexandre.igert@chemdef.fr (A.I.); virginia.guillon@chemdef.fr (V.G.); florian@nachon.net (F.N.); 2 European Synchrotron Radiation Facility, 71 Avenue des Martyrs, 38043 Grenoble CEDEX 9, France; gianluca.santoni@esrf.fr

**Keywords:** butyrylcholinesterase, prokaryotic expression, PROSS, SEC-MALS, differential scanning fluorimetry, 3D structure

## Abstract

Human butyrylcholinesterase is a performant stoichiometric bioscavenger of organophosphorous nerve agents. It is either isolated from outdated plasma or functionally expressed in eukaryotic systems. Here, we report the production of active human butyrylcholinesterase in a prokaryotic system after optimization of the primary sequence through the Protein Repair One Stop Shop process, a structure- and sequence-based algorithm for soluble bacterial expression of difficult eukaryotic proteins. The mutant enzyme was purified to homogeneity. Its kinetic parameters with substrate are similar to the endogenous human butyrylcholinesterase or recombinants produced in eukaryotic systems. The isolated protein was prone to crystallize and its 2.5-Å X-ray structure revealed an active site gorge region identical to that of previously solved structures. The advantages of this alternate expression system, particularly for the generation of butyrylcholinesterase variants with nerve agent hydrolysis activity, are discussed.

## 1. Introduction

Butyrylcholinesterase (BChE, EC 3.1.1.8) is the circulating homolog of acetylcholinesterase (AChE, EC 3.1.1.7), a membrane anchored enzyme responsible for the hydrolysis of the neurotransmitter acetylcholine in the inter-synaptic space in the central nervous system and neuromuscular junctions. While the crucial role of AChE for the efficient signal transduction has been demonstrated, the function of BChE remains unclear. However, its roles in decontaminating toxic circulating compounds such as nerve agents [1] or cocaine [2], hydrolyzing the “hunger” hormone ghrelin [3], as well as being a potential drug target candidate for late stage Alzheimer’s disease have been demonstrated [4,5].

Many studies on BChE have drawn benefit from biochemical and structural data obtained from recombinant human BChE that is produced in eukaryotic expression systems [6,7,8,9]. Despite their proven usefulness in the context of scientific research, these systems are particularly expensive for large scale productions, especially for the purification of human BChE as a bioscavenger against organophosphorous compounds (OP) intoxication [10]. It is estimated that 200 mg of pure human BChE is needed to protect an adult against 2 LD_50_ of OP toxic [11]. For this purpose, current large-scale purifications are realized from commercially produced Cohn fraction IV-4, from which gram quantities of protein [12,13] with remarkable pharmacokinetics properties [14,15] can be obtained. However, the annual plasma stocks would not allow enough production to cover the needs of a large population. On the other hand, recombinant productions in plants [16] or transgenic animals [17] allow for production of large protein quantities alleviating the stock issue but the protein isolated so far still require post-purification treatments [18], which raise the final cost of the injected dose to a yet unreasonable price.

A way to lower the dose of BChE required to afford sufficient protection is to identify mutants of BChE that are able to turn over nerve agent [19]. But despite some successes, this research has been slowed down by the difficulty to use directed evolution strategies for an enzyme that is only expressed in eukaryotic systems. Functional bacterial expression would open access to such directed evolution and potentially lower the production costs due to the inexpensiveness and scalability of the system. Attempts to express human BChE in *E. coli* were not successful, leading to isolation of the enzyme as non-refoldable inclusion bodies, certainly because of the presence of three disulfide bridges and nine *N*-glycosylation sites that enhances the stability and solubility of the enzyme [20]. Recently, Goldenzweig et al. have reported a structure- and sequence-based computational method for the design of stable proteins for bacterial expression, Protein Repair One Stop Shop (PROSS) [21]. Among the reported examples, the successful expression of human AChE was a breakthrough, as previous attempts to express human AChE in bacterial systems were also unsuccessful. The reported modified AChE contained 51 point mutations and was purified to homogeneity. This enzyme presented enzymatic parameters that are similar to wild-type enzyme and was prone to crystallize. We submitted human BChE to the PROSS server selecting specific residues to preserve its specificity and seven constructs were calculated. All of the proposed constructs were synthetized and screened for expression in an *E. coli* strain as thioredoxin fusion proteins. The clone presenting the highest BChE activity in the soluble fraction was further studied. The protein was purified and characterized enzymatically, biochemically, and structurally.

## 2. Results

### 2.1. PROSS Processing

Besides a structure of the target protein, the PROSS process requires the selection of specific residues in order to maintain important features, such as enzymatic activity and substrate specificity. For human BChE (hBChE), we selected residues of the active site triad (Ser198, Glu325, and His438), residues in close vicinity and along the gorge (Table 1).

**Figure 1:**
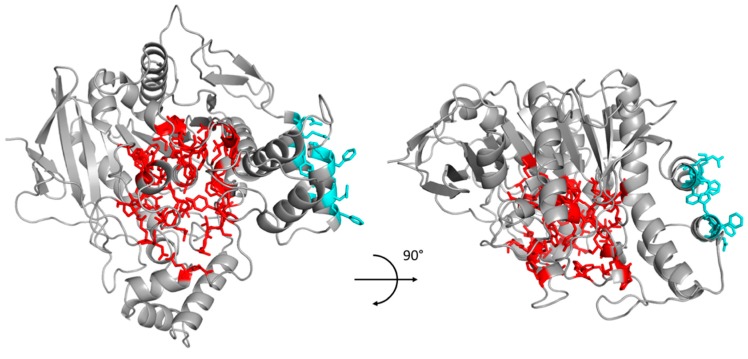
Structural representation of the selected conserved residues of human butyrylcholinesterase (hBChE) for the PROSS process. Residues conserved in order to preserve enzymatic activity are represented in red, those conserved to maintain dimer interaction are represented in cyan.

Moreover, in some reported BChE X-ray structures, the protein appears as a homodimer that is stabilized by a four-helix bundle, two pair of helices being provided by each monomer. To preserve this interaction, we also selected the residues involved in this dimer interface, namely residues 364, 367, 371, 372 in the first interacting helix and residues 517, 520, 521, 525, and 528 in the second interacting helix (Table 1). All of the fixed residues are illustrated in Figure 1.

The submitted PROSS process resulted in seven different constructs with 9 up to 47 distributed point mutations (Table 2) to test for bacterial expression (named hBChE-1 to 7), see Figure S1.

### 2.2. Constructs Cloning and Expression Screen

**Figure 2:**
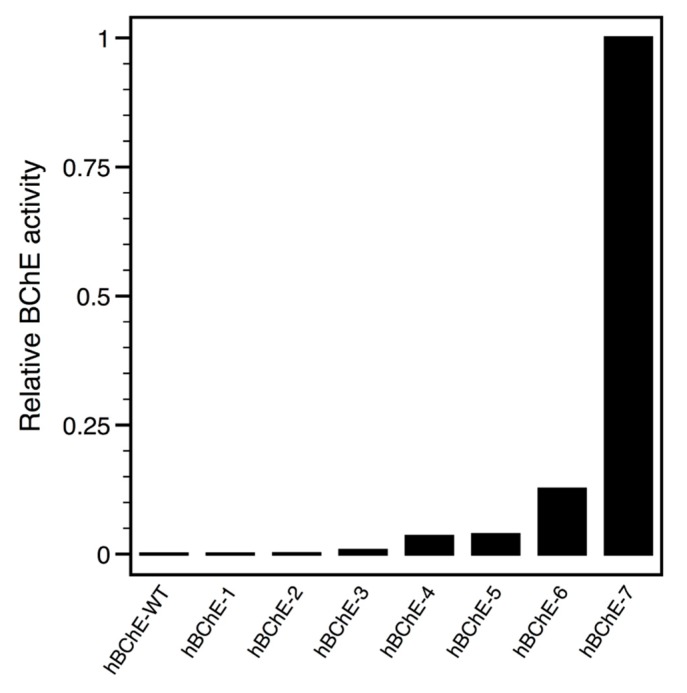
Comparison of BChE activity in the bacterial soluble extracts of the different BChE constructs. An aliquot on each soluble extract from pThioHis constructs of hBChE-wild-type (WT) and hBChE-1 to hBChE-7 was measured for butyrylthiocholine hydrolase activity. Activities were normalized to the highest measured sample.

Genes encoding for the seven PROSS-proposed constructs were synthetized using an *E. coli* optimized codon bias (see Figure S2) and sub-cloned into pThioHis vector for the expression of thioredoxin-hBChE fusion proteins, as previously reported for AChE [21]. Similarly, each construct was transformed into *E. coli* SHuffle^®^ T7 expression strain (New England Biolabs, Evry, France) that facilitates the expression of disulfide bridges containing proteins in the cytoplasm [22]. The initial expression screen consisted in the induction of protein expression by addition of isopropyl β-d-1-thiogalactopyranoside (IPTG) for 4 h at 37 °C of each construct from a minimal culture volume. Total soluble extracts obtained after cells lysis by sonication and centrifugation, were monitored for BChE activity (Figure 2).

While no BChE activity could be measured in the total soluble extracts of hBChE-wild-type (WT), hBChE-1, and hBChE-2 constructs, increasing activity appeared in the remaining constructs in correlation with the increasing number of mutations, and maximal BChE activity was detected for the hBChE-7 construct. Thus, we focused our efforts on the hBChE-7 construct for further characterization. We empirically determined best conditions for soluble protein expression by testing different production temperatures. To achieve this, induction was realized by IPTG addition on cells grown at OD_600nm_ = 0.8 and cells were cultured overnight at 37 °C or after shifting temperature down to 20 °C. BChE activity was assayed in the respective soluble extracts and the maximum activity was measured from cells grown at 37 °C (data not shown). However, we rapidly faced issues while trying to purify the resulting hBCHE-7 fusion protein. The recently developed BChE specific chromatography Hupresin [6] did not give satisfactory purity (data not shown). We switched to immobilized metal ion affinity chromatography (IMAC), as the thioredoxin (Trx) encoded in the pThioHis vector has been engineered to generate a histidine surface patch, but the fusion protein presented poor binding. We then decided to add a classical linear poly-histidine patch at the N-terminus of Trx by mutagenesis, and, in a second step, to replace the enterokinase cleaving site by the Tobacco Etch Virus (TEV) endopeptidase cleaving site for convenience and economic reason, as we routinely produce our recombinant poly-histidine-tagged TEV protease in the laboratory [23]. Upon generation of the resulting expression vector, p8HisTrxTEV-hBChE-7, we noticed a loss of production yield, from an estimated 5 mg down to about 1 mg per liter of Luria Broth (LB) medium. Nonetheless, we continued the production and purification steps using this construct.

### 2.3. Purification of hBChE-7

**Figure 3:**
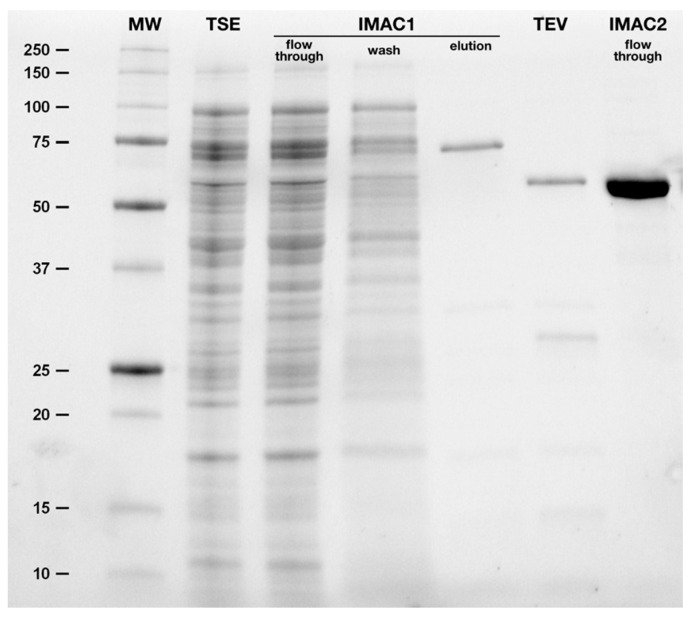
Analysis of the purification of hBCHE-7. A polyacrylamide gel electrophoresis was realized in denaturing conditions with an aliquot of different purification steps. Molecular weight (MW, and corresponding digits in kDa); total soluble extracts (TSE); flow-through, wash and elution fractions of the first metal ion affinity chromatography (IMAC1); dialysis and TEV-protease cleavage (TEV); and, flow-through fraction of the second IMAC (IMAC2).

For purification of the Trx-hBChE-7 fusion protein, total soluble extracts were prepared from 3 liters of LB culture (Figure 3, lane 2) and an initial IMAC step was realized.

After an extensive wash step (Figure 3, lane 4), the eluted fraction, containing BChE activity, was almost completely pure (Figure 3, lane 5). The eluted fraction was extensively dialyzed to remove excess imidazole simultaneously with the cleavage of the fusion protein by addition of TEV protease, characterized by the shift of the main band from about 75 kDa (calculated mass 74,112 Da) to 60 kDa (calculated mass 60,069 Da) (Figure 3, lane 6). Some protein precipitation was observed upon TEV cleavage. The second IMAC allowed isolation of the pure enzyme, devoid of Trx and TEV protease (Figure 3, lane 7). Protein loss was again observed upon protein concentration, however this simple 2-steps protocol allowed isolation of pure and homogenous hBChE-7, which was stored at 4 °C until further characterization.

### 2.4. Enzymatic Characterization

Due to their respective structures, human AChE and human BChE present specific enzymatic properties. The most remarkable is the difference of catalysis at high substrate concentrations where AChE is inhibited, while BChE is activated. We determined the kinetic parameters of the pure hBChE-7 using the modeled reported by Radić et al. [24]. (Table 3, Figure S3).

The BChE that is produced in the bacterial system presents a *K*_s_ similar to recombinant hBChE produced in chinese hamster ovary (CHO) cells [8] and by analogy to the plasmatic enzyme, while *K*_ss_ and *k*_cat_ parameters are slightly higher. Importantly, the *b* factor is similar to the one reported for the recombinant protein produced in CHO cells, pointing out that the specificity of BChE, i.e., activation at high substrate concentration, is maintained in BChE mutant.

### 2.5. Oligomeric State Characterization 

**Figure 4:**
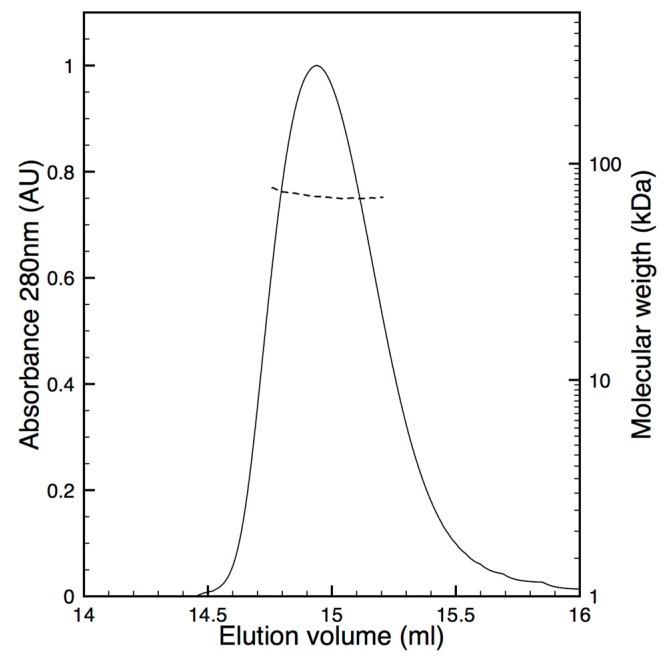
Oligomeric state analysis of pure hBChE-7. After separation of pure hBChE-7 on Superdex 200 Increase 10/300 equilibrated in 20 mM Tris pH 8.0, 150 mM NaCl, the major UV peak (plain line) was analyzed in line by multi-angles light scattering and a constant molecular weight of 70 + 2 kDa was measured (dashed line).

Physiologically BChE is mainly a tetramer through the interaction of the C-terminal helices of each monomer that is associated in a coiled coil motif around an antiparallel proline rich peptide originating from lamellipodin [26,27,28]. Some recombinant BChE productions and X-ray structures have reported the isolation of dimers of BChE and the tetrameric form can be view as a dimer of dimers. During the PROSS process we maintained the residues involved in this dimer interface, namely two helices, in the prospect to isolate a dimeric form of the recombinant enzyme. To address the oligomeric state of the isolated hBChE-7, a size-exclusion chromatography and multi-angles light scattering (SEC-MALS) analysis was carried out (Figure 4).

In the tested conditions a small fraction of the purified enzyme elutes as aggregated protein in the void volume and a second fraction as a major and monodisperse peak ($\frac{M_{w}}{M_{n}}\;$ = 1.001) around 15 mL elution. The molecular weight in this latter peak was determined at 70 ± 2 kDa, which is more in accordance with the isolation of a monomer (60 kDa) than a dimer (120 kDa).

### 2.6. Protein Stability and Further Stabilization

Goldenzweig et al. have reported that the PROSS processed human AChE presented a large thermal stabilization, with a melting temperature (Tm) shift from 44.0 to 66.2 °C, when measured in bacterial lysates. Similarly, for hBChE-7, we determined a Tm of 64.5 °C in bacterial lysates by monitoring residual activity after differential heating (data not shown). This high Tm was confirmed by screening multiple buffer compositions (pH, concentration and ionic force) by differential scanning fluorimetry (DSF) using pure hBChE-7. Upon dilution of pure enzyme in water, a Tm of 67.3 °C was determined and higher Tm (around 72 °C) were measured in 0.1 M buffers with pH ranging from 6.0 to 7.0 (KH_2_PO_4_, cacodylate, NaH_2_PO_4_). Further addition of 250 mM NaCl to these buffers resulted in a Tm increase to about 74 °C, denoting the stabilizing effect of the buffer ionic strength (Figure S4). A 20 µg/mL solution of hBChE-7 in 0.1 M HEPES pH 7.4, 0.25 M NaCl was prepared and stored at room temperature over more than two months and no evolution of the BChE activity was observed, apart from an initial low that could be accounted for protein sticking to the vial wall, denoting the high stability of the enzyme in these conditions (data not shown).

### 2.7. hBChE-7 Crystallization

**Figure 5:**
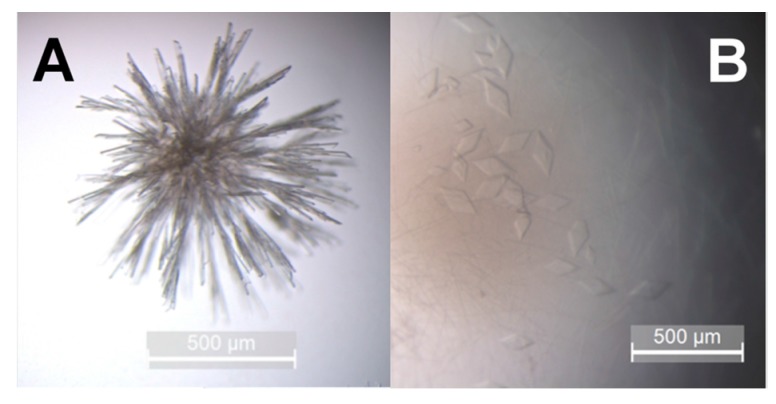
Crystals of hBChE-7. Crystallization was realized by the hanging drop method using 0.2 M NH_4_OAc pH 7.4 and 20% polyethylene glycol (PEG) 3350 as precipitation solution. hBhE-7 at a concentration of 2 mg mL^−1^ was mixed 2:1 with the precipitation solution. (**A**) crystals grown at 20 °C; (**B**) crystals grown at 4 °C.

Finally, we studied the crystallization propensity of the isolated hBChE-7 by using polyethylene glycol, a common precipitant used for cholinesterases crystallization, such as human [21,29], mouse [30], or *Torpedo californica* [31] AChE, and which was successfully used to crystallize human BChE [32]. Using these latter conditions, we obtained needle-shaped crystals overnight at 20 °C (Figure 5A), however the absence of clearly isolated crystals would hinder data collection. Such crystals probably result from a fast and uncontrolled crystallogenesis. We tried to slow-down the process by setting drops in the same conditions at 4 °C. This allowed isolation of single monoclinic diffracting crystals of about 200 µm in their largest dimension (Figure 5B).

### 2.8. hBChE-7 X-ray Structure

From crystals grown at 4 °C, complete datasets were collected and integrated in space group C121 with unit cell of a = 160 Å, b = 75 Å, c = 122 Å; α = 90°, β = 93°, γ = 90° with a resolution cutoff of 2.5 Å. Despite the presence of a translational non-crystallographic symmetry, molecular replacement procedure was successful. We found two monomers in the asymmetric unit and statistics for the refined model are presented in Table 4.

**Figure 6:**
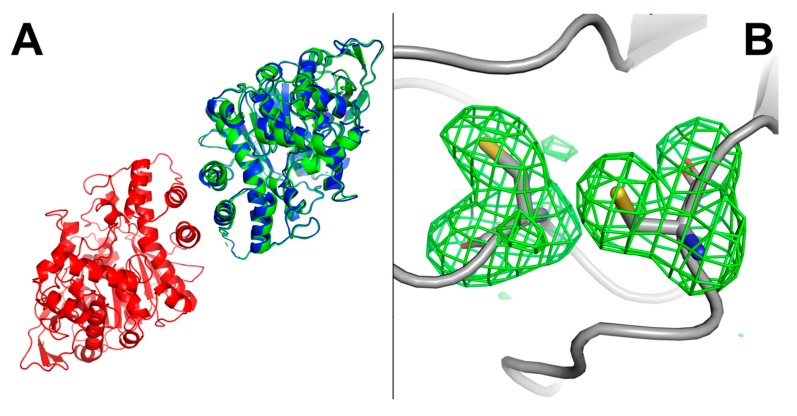
Structure of hBChE-7. (**A**) cartoon representation of the *in cristallo* dimer form of hBChE-7 (PDB ID 6EMI) as red and blue chains. Superposition of the structure of recombinant hBChE from CHO cells (PDB ID 1P0I), green chain; (**B**) representation of the broken Cys65-Cys92 disulfide bond in chain B. Protein backbone is represented in gray. Residues Cys65 and Cys92 are represented as sticks with carbon atoms in gray, oxygen atoms in red, nitrogen atoms in blue and sulfide atoms in yellow. An |*F*_o_ − *F*_c_| electron density map calculated by omitting residues Cys65 and Cys92 from the model is represented as a green mesh with a 3.0 σ contour.

The overall structure is very similar to previously recombinant human BChE solved structure (RMSD = 0.452 Å). Analysis of the crystal packing showed that each monomer of the asymmetric unit forms the canonical cholinesterase dimer with a symmetric mate through the four-helix bundle formed by the 362–373 and 516–529 helices of each monomer (Figure 6A), as previously observed [6].

**Figure 7:**
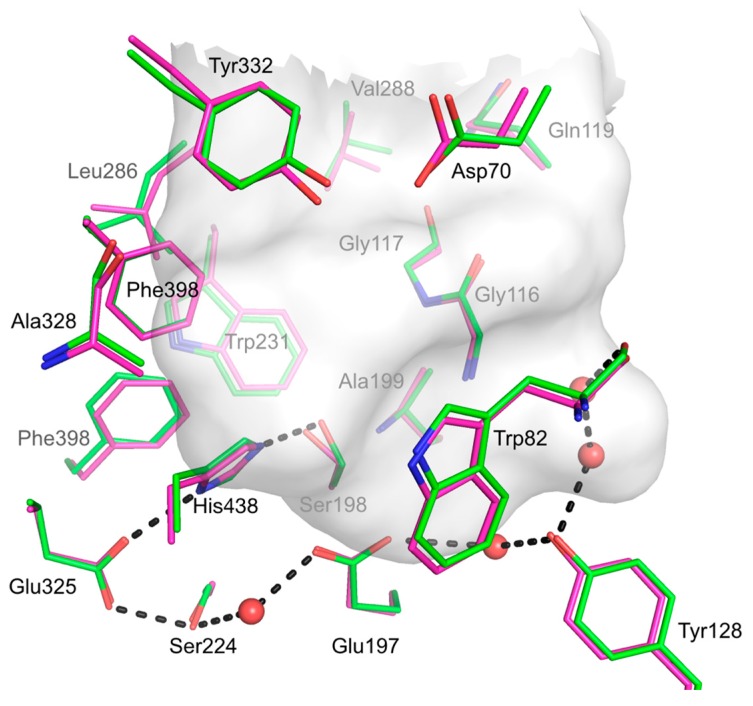
Active site gorge comparison of hBChE-7 and recombinant hBChE produced in CHO cells. Residues forming the active site triad (Ser198, Glu335, and His438) and those forming the active-site gorge are represented as sticks. Carbon atoms are represented in green and magenta for hBChE-7 structure (PDB ID 6EMI) and hBChE expressed in CHO cells (PDB ID 1P0I), respectively. Nitrogen atoms are represented in blue and oxygen atoms in red. Water molecules are represented as red spheres. The active site gorge is represented as a semi-transparent grey surface.

The respective active site entrances are thus facing the opposite faces of the dimer. Active site residues and residues forming the gorge adopt very similar conformations when compared to respective residues from the structure of recombinant human BChE from CHO cells. Apart from the mutated residues resulting from the PROSS process, the main difference between the structure of human BChE produced in *E. coli* and the structures of human BChE produced in eukaryotic systems (CHO cells or insect cells) lies in the fate of the Cys65–Cys92 disulfide bond. Taking account residue occupancy, this bond is partially broken in chain A (about 30%) and totally in chain B (Figure 6B). Data were collected at low effective dose of 0.25 MGy (calculated with RADDOSE3D [33]), thus it is difficult to consider this weak bond as a consequence of radiation damage. On contrary, the two other disulfide bonds, Cys252–Cys263 and Cys400–Cys519, remain formed for both chains. Interestingly despite the partial formation of the Cys65–Cys92 disulfide bond, the overall structure remains unaffected in particular for the active site gorge region (see Figure 7).

However, the Cys65–Cys92 bond may be important for the stabilization of the Ω loop and the interaction between Asp70 and Tyr332 residues that form part of the BChE peripheral subsite [34,35]. However, molecular dynamics (50 ns, amber99sb force field, at 300 K, in tip4p waters) of hBCHE-7 with reduced Cys65–Cys92 disulfide bond did not show any sign of denaturation compared to the oxidized one (data not shown).

## 3. Discussion

Our decade efforts to produce human BChE in a prokaryotic system, and to our best knowledge, efforts of other groups worldwide, were unsuccessful. Generally, the difficulty to obtain soluble and active eukaryotic proteins is potentially due to the presence of disulfide bridges, which upon wrong formation leads to protein misfolding and isolation as inclusion bodies. However, presence of three disulfide bridges in BChE does not explain entirely the prokaryotic expression difficulties, as the WT construct reported here does not express as active enzyme in the *E. coli* SHuffle strain, which is optimized for disulfide bond generation. Post-translational modifications such as *N*-glycosylation are also an important factor for protein stability. Even in a eukaryotic system, generation of low glycosylated forms of human BChE limited the expression [8]. Nevertheless, the optimization of the surface residues and protein stabilization proposed by the PROSS algorithm, allowed for the isolation of active BChE in the soluble extracts, which was then purified to homogeneity. It is important to note that all of the potential disulfide bonds of wild-type BChE remain in all the PROSS proposed constructs, stressing the importance of the PROSS stabilization process. Besides the low production yields that hinder economical large-scale production for such therapeutic protein, the pure hBChE mutant behaves similarly to the wild-type enzyme on the kinetic point of view, with an activation in presence of excess of substrate. This is in accordance with the conservation of Asp70 residue during the PROSS process, which is crucial for this phenomenon [36].

Regarding oligomerization, the isolated protein behaves as a monomer in solution when injected at 1 mg/mL for SEC-MALS analysis despite the conservation of residues involved in dimer interface. The presence of two mutations in close vicinity of the very last helix, Gln518His and Thr523Asn, may destabilize this latter and then impair dimer formation. However, the crystal structure reveals the dimeric form certainly driven by the higher and increasing local protein concentration. In physiological conditions, BChE is mainly a tetramer and this oligomerization is triggered by the interaction of a proline-rich peptide and the C-terminal domain of BChE, which is deleted in the here-reported construct. Co-expression of a proline-rich peptide has been successfully reported to promote isolation of BChE tetramers in eukaryotic systems [9]. We are now focusing our efforts on developing such a co-expression strategy in our prokaryotic system as it could result to a more stable and better therapeutic enzyme. Indeed, studies have shown that BChE tetramers present better pharmacokinetic profiles than monomers or dimers [37]. BChE is also a highly *N*-glycozylated enzyme that affects its long circulation half-life, but such modifications do not occur in the reported prokaryotic host. Chemical modifications may be necessary to improve the lifetime and attenuate possible immune response such as PEGylation that has been successfully used for recombinant BChE that is produced in plants or transgenic animals or other therapeutic proteins [38]. If the pharmacokinetic properties turn out to be not satisfactory enough to use this enzyme as a prophylactic treatment, such expression system, could allow the generation of an affordable enzyme-based nerve agent decontaminant for skin or delicate equipment, for which the use of harsh conditions (sodium hydroxide or sodium hypochlorite) is not possible.

Probably a major point of interest of this prokaryotic expression system is its possible use as a tool for high-throughput screening of BChE mutants for the development of catalytic bioscavengers, such as what have been done with bacterial phosphotriesterases [39]. Indeed, BChE variants, such as the Gly117His and Gly117His/Glu197Gln human BChE mutants [40,41] or swine BChE (manuscript in preparation) can catalyze OP hydrolysis. Such an expression system will allow for generation and screening of large BChE mutant libraries for OP hydrolysis properties. Isolation of a highly efficient mutant will then decrease the amount of required enzyme to afford efficient protection against OP intoxication, and then mechanically lower the effective cost of the therapeutic dose. Other BChE-based projects could benefit from this prokaryotic expression system, such as the production of the BChE variant that is developed to improve cocaine hydrolysis [42] and the possibility to obtain structural data from this recombinant protein will ease the development of BChE-specific reactivators [43] or for the study of anti-Alzheimer’s molecules [44].

## 4. Materials and Methods

### 4.1. PROSS Processing

The PROSS job was submitted online on the dedicated web server (http://pross.weizmann.ac.il) using wild-type human BChE sequence (GenBank AAH18141.1) and the pdb file supplied in supporting information. This file, prepared from PDB ID 4AQD, corresponds to the BChE homodimer which long C-terminal helix promoting tetramerization was truncated. Post-translational modifications (*N*-glycosylations), water molecules and other ligands were omitted. Chain A of the submitted pdb file was selected for the PROSS process along with specific residues to maintain both enzymatic activity and dimer interface (Table 1). The required multiple sequences alignment was automatically generated by the PROSS process using default parameters.

### 4.2. BChE Constructs 

The seven constructs proposed by the PROSS process were synthetized using bacterial optimized codons (GeneArt, ThermoFischer Scientific, Courtaboeuf, France). Constructs were sub-cloned between KpnI and XhoI sites (New England Biolabs, Evry, France) of the pThioHis (ThermoFischer Scientific, Courtaboeuf, France) vector for the expression of thioredoxin-BChE fusion proteins and allowing for purification by metal chelate affinity and enterokinase cleavage for Trx removal. Addition of an octa-histidine tag at the N-terminal position was realized by site-directed ligase independent mutagenesis [45] using the pThioHis-hBChE-7 vector as template, Q5^®^ High-Fidelity DNA polymerase (New England Biolabs, Evry, France) and the two pairs of oligonucleotides: 8His-F1/8His-F2 and 8His-R1/8His-R2 (Eurogentec, Angers, France) (Table 5), leading to p8HisThio-hBCHE-7.

Replacement of the enterokinase cleaving site by the Tobacco Etch Virus endopeptidase (TEV) site was carried out similarly from p8HisThio-hBCHE-7 as template and the following two pairs of oligonucleotides: TEV-F1/TEV-F2 and TEV-R1/TEV-R2 (Eurogentec, Angers, France) (Table 5). The NdeI/XhoI fragment coding for the whole fusion enzyme was sub-cloned back into fresh pThioHis vector that never had any mutagenesis round, leading to p8HisThioTEV-hBChE-7 expression vector.

### 4.3. Expression and Purification

For the initial expression screening, the different expression vectors (pThioHis-hBChE-1 to pThioHis-hBChE-7) were independently transformed into SHuffle^®^ T7 Competent *E. coli* (New England Biolabs, Evry, France). Clones of each construct were cultured overnight in 5 mL of LB (Sigma Aldrich, Saint-Quentin-Fallavier, France), supplemented by 100 µg mL^−1^ ampicillin (Sigma Aldrich, Saint-Quentin-Fallavier, France) at 37 °C, and protein synthesis was induced by addition of 1 mM IPTG (Acros Organics) for 4 h at 37 °C. Pelleted cells were suspended in 2 mL of 20 mM Tris pH 7.5, 150 mM NaCl (Sigma Aldrich, Saint-Quentin-Fallavier, France). Total soluble extracts were prepared by pulsed sonication on ice for 3 times 5 min using a S2 micro-probe (2 mm) equipped UP200S sonicator (Hielscher, Teltow, Germany) and centrifugation at 20,000× *g* for 20 min at 4 °C. For large scale production and purification, p8HisThioTEV-hBChE-7 vector was transformed into SHuffle^®^ T7 Competent *E. coli* and 3 L of LB were prepared after IPTG induction at OD_600nm_ = 0.8 and overnight culture at 37 °C. Bacterial pellet was suspended in 150 mL of 20 mM HEPES pH 7.5, 0.3 M NaCl, 15 mM Imidazole and sonicated on ice for 5 times 3 min with 0.5 s pulses using a 12.5 mm probe equipped Sonic Ruptor 400 (Omni, Kennesaw, GA, USA). After centrifugation at 35,000× *g* for 30 min at 4 °C, the total soluble extract was loaded onto 7 mL of cOmplete^TM^ His-Tag resin (Roche, Boulogne-Billancourt, France) packed in a XK 16/20 column (GE Healthcare, Vélizy-Villacoublay, France) and equilibrated in 20 mM HEPES pH 7.5, 0.3 M NaCl, 15 mM Imidazole (Buffer A). After loading, the column was extensively washed in Buffer A before step elution with 20 mM HEPES pH 7.5, 0.3 M NaCl, 0.3 M Imidazole. Fractions containing maximum BChE activity were pooled and digested with recombinant TEV protease during overnight dialysis (Slide-A-Lyzer cassette 10 kDa MWCO, ThermoFischer Scientific, Courtaboeuf, France) against 4 L of 20 mM HEPES pH 7.5, NaCl 0.1 M (Buffer B). The solution was then reloaded onto the same 7 mL cOmplete^TM^ column equilibrated in Buffer B, washed in Buffer B and the flow through fraction, containing BChE activity, was collected and concentrated (Centricon Plus-70 30 kDa MWCO, Millipore, Molsheim, France)*.* Concentrated enzyme was conserved at 4 °C before enzymatic and biophysical characterization. Aliquots of the different steps were analyzed on acrylamide gel electrophoresis in denaturing conditions (Any kD Mini-PROTEAN TGX precast gel, Biorad, Marnes-la-Coquette, France) to assess protein purification.

### 4.4. BChE Activity

BChE enzymatic activity was followed using the modified Ellman’s method [46] and butyrylthiocholine (BTC) (Sigma Aldrich, Saint-Quentin-Fallavier, France) as protein substrate. Briefly, enzyme solution was assayed in 1 mL of 0.1 M phosphate buffer pH 7.4, 0.1 g L^−1^ 5-5′-dithiobis(2-nitrobenzoic acid) (Sigma Aldrich, Saint-Quentin-Fallavier, France) and 1 mM BTC. Kinetics were monitored at 412 nm on a Cary^®^ 50 spectrophotometer (Agilent Technologies, Courtaboeuf, France) at room temperature. For enzymatic parameters determination, pure enzyme was diluted in 20 mM HEPES pH 7.4, 150 mM NaCl, 0.1% bovine serum albumin (Sigma Aldrich, Saint-Quentin-Fallavier, France) to ensure maximal stability and activity was measured over a BTC concentration ranging from 1 µM to 7 mM and parameters were obtained by nonlinear fitting with proFit v7 (Quantum Soft, Uetikon am See, Switzerland) of the apparent rate vs. substrate concentration using the model proposed by Radić for cholinesterases [24] and characterized by the following equation:

$$v=\frac{v_{\max}\;\times \;\left[S\right]}{K_{s}+\left[S\right]}\times \frac{K_{\mathrm{ss}}+b\left[S\right]}{K_{\mathrm{ss}}+\left[S\right]}$$

where v is the apparent rate, *K*s is the substrate dissociation constant at the acylation site, *K*ss the substrate dissociation constant at the peripheral site, and *b*, the factor affecting *v*_max_ during the binding of a substrate molecule to the peripheral site. *k*_cat_ was calculated from *v*_max_ using an extinction coefficient of 14,150 M^−1^ cm^−1^ for thionitrobenzoate and determination of active site concentration by titration of the residual activity using the achiral organophosphorous inhibitor echothiophate, as previously reported [8]. On daily routine, pure protein concentration was determined spectrophotometrically using a calculated $A_{280nm}^{0.1\%}=1.67$.

### 4.5. SEC-MALS Analysis 

For oligomerization state determination, 50 µL of pure hBChE-7 at a concentration of 1 mg mL^−1^ was injected onto a Superdex-200 Increase 10/300 column (GE Healthcare, Vélizy-Villacoublay, France) developed in 20 mM Tris pH 8.0, 150 mM NaCl at a flow rate of 0.5 mL min^−1^ on an AKTA purifier (GE Healthcare, Vélizy-Villacoublay, France). Multi-angle laser light scattering was recorded in-line with an 8 angles DAWN^®^ Heleos^®^ II detector (Wyatt Technologies Corp., Toulouse, France) using a 663 nm laser light and refractive index measurements were recorded on an Optilab^®^ T-rEx differential refractometer (Wyatt Technologies Corp., Toulouse, France). Data were treated using the Astra^®^ v6.1 software (Wyatt Technologies Corp., Toulouse, France), and the average molecular weight was determined using a differential index of refraction ($\frac{dn}{dc}$) value of 0.185.

### 4.6. Differential Scanning Fluorimetry Assay

Tm determination in different buffer conditions was realized by DSF using a home-made buffer screen inspired by the one reported by Boivin et al. [47]. Briefly, 2 µL of pure hBChE-7 at 1 mg mL^−1^ was added into a 96-wells PCR plate containing 21 µL of each buffer condition of the screen (see composition in SI-5) and 2 µL of a 50× SYPRO Orange dye (ThermoFischer Scientific, Courtaboeuf, France) solution in water was finally added. The 96-wells PCR plate was sealed and centrifuged to avoid any air bubbles and a melting curve from 25 to 99 °C was realized on a StepOne Plus Real-Time PCR system (ThermoFisher Scientific, Courtaboeuf, France). Data were analyzed using the DSF Analysis Tools v3.0.2 developed by Dr. Franck Niesen (Oxford, UK, ftp://ftp.sgc.ox.ac.uk/pub/biophysics/) and Tm determined by fitting the Boltzmann equation using Prism v6.0 (GraphPad Software, San Diego, CA, USA).

### 4.7. Crystallogenesis, X-ray Data Collection and Analysis

Crystallization was realized using the hanging drop method at 20 °C and 4 °C by mixing 3 µL of pure hBChE-7 at 2 mg mL^−1^ with 1.5 µL of 0.2 M NH_4_OAc pH 7.4, 20% PEG 3350 as precipitating solution. Crystals obtained at 4 °C were cryoprotected in precipitating solution containing 20% glycerol, before flash cooling into liquid nitrogen. Diffraction data were collected on ID29 beamline [48] at the European Synchrotron Research Facility (ESRF, Grenoble, France) under nitrogen gas stream at 100 K. Dataset were processed with XDS [49] by the ESRF automatic data processing pipeline [50], intensities of integrated reflections were scaled using XSCALE and converted to ccp4 compatible mtz format through POINTLESS, TRUNCATE, and SCALA. Structure was solved by molecular replacement using Phaser from the PHENIX suite [51] and recombinant human BChE model (PDB ID 1P0I) devoid of water, glycan, and other ligand molecules. Initial model was refined by iterative cycles of model building in Coot [52] and restrained refinements with Phenix refine. The protein structure was illustrated using the PyMOL software v2 (Schrödinger, LLC, Cambridge, MA, USA) and the HOLLOW software [53] for the gorge surface. Structural data have been submitted to the Protein Data Bank under accession number 6EMI.

## Figures and Tables

**Table 1:** Conserved residues selected during the Protein Repair One Stop Shop (PROSS) process in order to preserve both enzymatic activity and dimer interface. Residues of the catalytic triad are represented in bold. Positions are numbered from the physiologically maturated protein.

Enzymatic Activity	Dimer Interface
67, 68, 70, 77, 81, 82,83, 84, 112, 114, 115, 116, 117, 118, 119, 120, 128, 146, 197, **198**, 199, 224, 231, 276, 277, 285, 286, 287, 288, 289, 322, **325**, 328, 329, 332, 398, 430, 433, 434, 437, **438**, 439, 440, 441, 442	364, 367, 371, 372, 517, 520, 521, 525, 528

**Table 2:** Mutations number and positions of the seven human BChE constructs generated by the PROSS process. Positions are numbered from the physiologically maturated protein.

Name	Mutations Number	Mutation Positions
**hBChE-1**	9	7, 48, 54, 215, 250, 397, 454, 466, 468
**hBChE-2**	15	7, 48, 54, 62, 110, 215, 236, 237, 250, 397, 406, 412, 454, 466, 468
**hBChE-3**	18	7, 48, 54, 110, 126, 180, 215, 236, 237, 250, 274, 379, 397, 406, 412, 454, 466, 468
**hBChE-4**	21	7, 48, 54, 110, 126, 180, 215, 227, 236, 237, 250, 274, 360, 379, 397, 406, 412, 454, 466, 468, 469
**hBChE-5**	25	7, 48, 54, 110, 111, 126, 180, 215, 227, 236, 237, 250, 274, 360, 379, 397, 406, 409, 412, 454, 466, 468, 469, 489, 523
**hBChE-6**	36	7, 48, 54, 66, 71, 110, 111, 126, 176, 180, 215, 227, 234, 236, 237, 250, 274, 305, 356, 360, 377, 379, 380, 391, 397, 406, 409, 412, 417, 454, 466, 468, 469, 489, 518, 523
**hBChE-7**	47	7, 48, 54, 66, 71, 110, 111, 126, 176, 180, 188, 190, 191, 215, 227, 234, 236, 237, 250, 274, 283, 305, 342, 356, 360, 377, 379, 380, 387, 390, 391, 397, 406, 409, 410, 412, 417, 454, 459, 466, 468, 469, 489, 495, 508, 518, 523

**Table 3:** Comparison of the determined enzymatic parameters of the hBChE-7 mutant. *K*_s_ is the dissociation constant at the catalytic site, *K*_ss_ is the dissociation constant at the peripheral non-productive site, *b* is the factor affecting *k*_cat_ describing inhibition or activation resulting from the binding of substrate at the peripheral site. Parameters are compared to those reported to hBChE-4SugOff produced in Chinese Hamster Ovary (CHO) cells and human plasma BChE.

	*K*_s_ (µM)	*K*_ss_ (µM)	*b*	*k*_cat_ (min^−1^)
hBChE-7	30.0 ± 2.5	1291 ± 112	2.80 ± 0.10	46,715
hBChE_CHO_ [8]	25.6 ± 0.4	510 ± 35	2.85 ± 0.15	28,000
hBChE_plasma_ [25]	20	300	2.4	24,000

**Table 4:** X-ray data collection and refinement statistics for hBChE-7 structure. R-work = Σ |*F*_o_| − |*F*_c_|/Σ |*F*_o_|, *F*_o_ and *F*_c_ are observed and calculated structure factors, R-free set uses 5% of randomly chosen reflections. Statistics for the highest-resolution shell are shown in parentheses.

	hBChE-7
*Data collection*	
X-ray source—beamline	ESRF—ID29-1
Wavelength (Å)	1.074
Resolution range (Å) (highest resolution shell)	65.02–2.476 (2.565–2.476)
Space group	C 1 2 1
Unit cell parameters (Å) °	159.9 75.1 122.1 90.0 93.4 90.0
Total reflections	175,981 (17,785)
Unique reflections	51,072 (5027)
Multiplicity	3.4 (3.5)
Completeness (%)	98.37 (97.86)
Mean I/sigma	7.92 (2.30)
Wilson B-factor	36.13
R-merge	0.1163 (0.5303)
R-meas	0.1381 (0.6246)
R-pim	0.0737 (0.3278)
CC_1/2_	0.995 (0.887)
CC *	0.999 (0.969)
*Refinement statistics*	
Reflections used in refinement	50,925 (5021)
Reflections used for R-free	2574 (241)
R-work	0.2219 (0.2937)
R-free	0.2553 (0.3305)
CC (work)	0.956 (0.880)
CC (free)	0.947 (0.831)
Number of non-hydrogen atoms	9018
Macromolecules	8423
Ligands	219
Solvent	376
Protein residues	1054
RMS (bonds)	0.003
RMS (angles)	0.54
Ramachandran favored (%)	95.62
Ramachandran allowed (%)	4.00
Ramachandran outliers (%)	0.38
Clashscore	5.66
Average B-factor	41.57
macromolecule	41.08
ligands	56.33
solvent	44.16

ESRF: European Synchrotron Radiation Facility, CC: Pearson’s correlation coefficient statistics.

**Table 5:** Oligonucleotides used for mutagenesis experiments in this study.

Name	Sequence
8His-F1	5′-CATCACCATCACCATCACCATCACTCTGATAAAATTATTCATCTG-3′
8His-F2	5′-TCTGATAAAATTATTCATCTG-3′
8His-R1	5′-GTGATGGTGATGGTGATGGTGATGCATATGTATATACCTCTTTAA-3′
8His-R2	5′-CATATGTATATACCTCTTTAA-3′
TEV-F1	5′-GAGAATCTTTATTTTCAGGGCGCCATGGAAGATGACATTATCATC-3′
TEV-F2	5′-CCATGGAAGATGACATTATCATC-3′
TEV-R1	5′-CGCCCTGAAAATAAAGATTCTCACCGGATCCAGAGCCGGCCAG-3′
TEV-R2	5′-ACCGGATCCAGAGCCGGCCAG-3′

## Supplementary Materials

The following are available online. Figure S1: Primary sequence alignment of wild-type human BChE and the seven PROSS proposed constructs, Figure S2: Bacterial optimized sequences of the 7 constructs proposed by the PROSS process, Figure S3: Determination of kinetic parameters of hBChE-7 for BTC hydrolysis, Figure S4: Effects of different buffers on hBChE-7 thermal stability. Figure S5: Composition of the buffer screen used in the DSF assay, dimer.pdb: structure of human BChE dimer used for the PROSS job submission.
