# Supplementary material for: Bacterial Expression of Human Butyrylcholinesterase as a Tool for Nerve Agent Bioscavengers Development
Source: Molecules. 2017 Oct 27;22(11):1828. doi: 10.3390/molecules22111828 (PMC6150354; doi:10.3390/molecules22111828)
Supplement: Supplementary file 1 [file molecules-22-01828-s001.pdf]

SUPPORTING INFORMATION

Bacterial Expression of Human Butyrylcholinesterase as a Tool for Nerve Agent Bioscavengers Development.

Xavier Brazzolotto [1,*], Alexandre Igert [1], Virginia Guillon [1], Gianluca Santoni [2], Florian Nachon [1]

[1] Institut de Recherche Biomédicale des Armées, Département de Toxicologie et Risques Chimiques, BP73, 91223, Brétigny sur Orge, France

[2] European Synchrotron Radiation Facility, 71 Avenue des Martyrs, 38043, Grenoble Cedex 9, France

* Corresponding author

CORRESPONDING AUTHOR:

Xavier Brazzolotto, PhD

Institut de Recherche Biomédicale des Armées, Département de Toxicologie et Risques Chimiques

1, Place Général Valérie André, BP 73, 91223, Brétigny-sur-Orge, France

Tel (+33) 1 78 65 14 00

Fax (+33) 1 78 65 14 58

Email: xavier@brazzolotto.net

Figure S1. Primary sequence alignment of wild-type human BChE and the seven *PROSS* proposed constructs. *PROSS* conserved residues are represented in red for the maintain of BChE activity and in cyan for the dimer interface. Mutated residues are highlighted in grey. Positions are numbered from the physiologically maturated protein.

```
hBCHE-WT  1     EDDIIIATKNGKVRGMNLTVFGGTVTAFLGIPYAQPPLGRLRFKKPQSLTKWSDIWNATKYANSCCQNIDQSFPGFHGSEMWNPNTDLSEDCLYLNVWIPAPKPKNATV  109
hBCHE-1   0    MEDDIIITTKNGKVRGMNLTVFGGTVTAFLGIPYAQPPLGRLRFKKPQPLTKWSGIWNATKYANSCCQNIDQSFPGFHGSEMWNPNTDLSEDCLYLNVWIPAPKPKNATV  109
hBCHE-2   0    MEDDIIITTKNGKVRGMNLTVFGGTVTAFLGIPYAQPPLGRLRFKKPQPLTKWSGIWNATKYPNSCCQNIDQSFPGFHGSEMWNPNTDLSEDCLYLNVWIPAPKPKNATV  109
hBCHE-3   0    MEDDIIITTKNGKVRGMNLTVFGGTVTAFLGIPYAQPPLGRLRFKKPQPLTKWSGIWNATKYANSCCQNIDQSFPGFHGSEMWNPNTDLSEDCLYLNVWIPAPKPKNATV  109
hBCHE-4   0    MEDDIIITTKNGKVRGMNLTVFGGTVTAFLGIPYAQPPLGRLRFKKPQPLTKWSGIWNATKYANSCCQNIDQSFPGFHGSEMWNPNTDLSEDCLYLNVWIPAPKPKNATV  109
hBCHE-5   0    MEDDIIITTKNGKVRGMNLTVFGGTVTAFLGIPYAQPPLGRLRFKKPQPLTKWSGIWNATKYANSCCQNIDQSFPGFHGSEMWNPNTDLSEDCLYLNVWIPAPKPKNATV  109
hBCHE-6   0    MEDDIIITTKNGKVRGMNLTVFGGTVTAFLGIPYAQPPLGRLRFKKPQPLTKWSGIWNATKYANSCMQNIDTSFPGFHGSEMWNPNTDLSEDCLYLNVWIPAPKPKNATV  109
hBCHE-7   0    MEDDIIITTKNGKVRGMNLTVFGGTVTAFLGIPYAQPPLGRLRFKKPQPLTKWSGIWNATKYANSCMQNIDTSFPGFHGSEMWNPNTDLSEDCLYLNVWIPAPKPKNATV  109

hBCHE-WT  110  LIWIYGGGFQTGTSSLHVYDGKFLARVERVIVVSMNYRVGALGFLALPGNPEAPGNMGLFDQQLALQWVQKNIAAFGGNPKSVTLFGESAGAASVSLHLLSPGSHSLFTR  219
hBCHE-1   110  LIWIYGGGFQTGTSSLHVYDGKFLARVERVIVVSMNYRVGALGFLALPGNPEAPGNMGLFDQQLALQWVQKNIAAFGGNPKSVTLFGESAGAASVSLHLLSPGSHPLFTR  219
hBCHE-2   110  MIWIYGGGFQTGTSSLHVYDGKFLARVERVIVVSMNYRVGALGFLALPGNPEAPGNMGLFDQQLALQWVQKNIAAFGGNPKSVTLFGESAGAASVSLHLLSPGSHPLFTR  219
hBCHE-3   110  MIWIYGGGFQTGTSSLPVYDGKFLARVERVIVVSMNYRVGALGFLALPGNPEAPGNMGLFDQQLALQWVQDNIAAFGGNPKSVTLFGESAGAASVSLHLLSPGSHPLFTR  219
hBCHE-4   110  MIWIYGGGFQTGTSSLPVYDGKFLARVERVIVVSMNYRVGALGFLALPGNPEAPGNMGLFDQQLALQWVQDNIAAFGGNPKSVTLFGESAGAASVSLHLLSPGSHPLFTR  219
hBCHE-5   110  MVWIYGGGFQTGTSSLPVYDGKFLARVERVIVVSMNYRVGALGFLALPGNPEAPGNMGLFDQQLALQWVQDNIAAFGGNPKSVTLFGESAGAASVSLHLLSPGSHPLFTR  219
hBCHE-6   110  MVWIYGGGFQTGTSSLPVYDGKFLARVERVIVVSMNYRVGALGFLALPGNPEAPGNMGLFDQQLALKWVQDNIAAFGGNPKSVTLFGESAGAASVSLHLLSPGSHPLFTR  219
hBCHE-7   110  MVWIYGGGFQTGTSSLPVYDGKFLARVERVIVVSMNYRVGALGFLALPGNPEAPGNMGLFDQQLALKWVQDNIAAFGGDPNRVTLFGESAGAASVSLHLLSPGSHPLFTR  219

hBCHE-WT  220  AILQSGSFNAPWAVTSLYEARNRTLNLAKLTGCSRENETEIIKCLRNKDPQEILLNEAFVVPYGTPLSVNFGPTVDGDFLTDMPDILLELGQFKKTQILVGVNKDEGTAF  329
hBCHE-1   220  AILQSGSFNAPWAVTSLYEARNRTLNLAKLLGCSRENETEIIKCLRNKDPQEILLNEAFVVPYGTPLSVNFGPTVDGDFLTDMPDILLELGQFKKTQILVGVNKDEGTAF  329
hBCHE-2   220  AILQSGSFNAPWAVTSPEEARNRTLNLAKLLGCSRENETEIIKCLRNKDPQEILLNEAFVVPYGTPLSVNFGPTVDGDFLTDMPDILLELGQFKKTQILVGVNKDEGTAF  329
hBCHE-3   220  AILQSGSFNAPWAVTSPEEARNRTLNLAKLLGCSRENETEIIKCLRNKDPQEILDNEAFVVPYGTPLSVNFGPTVDGDFLTDMPDILLELGQFKKTQILVGVNKDEGTAF  329
hBCHE-4   220  AILQSGSANAPWAVTSPEEARNRTLNLAKLLGCSRENETEIIKCLRNKDPQEILDNEAFVVPYGTPLSVNFGPTVDGDFLTDMPDILLELGQFKKTQILVGVNKDEGTAF  329
hBCHE-5   220  AILQSGSANAPWAVTSPEEARNRTLNLAKLLGCSRENETEIIKCLRNKDPQEILDNEAFVVPYGTPLSVNFGPTVDGDFLTDMPDILLELGQFKKTQILVGVNKDEGTAF  329
hBCHE-6   220  AILQSGSANAPWAVMSPEEARNRTLNLAKLLGCSRENETEIIKCLRNKDPQEILDNEAFVVPYGTPLSVNFGPTVDGDFLTDMPDTLLELGQFKKTQILVGVNKDEGTAF  329
hBCHE-7   220  AILQSGSANAPWAVMSPEEARNRTLNLAKLLGCSRENETEIIKCLRNKDPQEILDNEAFVVPYSTPLSVNFGPTVDGDFLTDMPDTLLELGQFKKTQILVGVNKDEGTAF  329

hBCHE-WT  330  LVYGAPGFSKDNNSIITRKEFQEGLKIFFPGVSEFGKESILFHYTDWVDDQRPENYREALGDVVGDYNFICPALEFTKKFSEWGNNAFFYYFEHRSSKLPWPEWMGVMHG  439
hBCHE-1   330  LVYGAPGFSKDNNSIITRKEFQEGLKIFFPGVSEFGKESILFHYTDWVDDQRPENYREALGDVVGDYFFICPALEFTKKFSEWGNNAFFYYFEHRSSKLPWPEWMGVMHG  439
hBCHE-2   330  LVYGAPGFSKDNNSIITRKEFQEGLKIFFPGVSEFGKESILFHYTDWVDDQRPENYREALGDVVGDYFFICPALEFAKKFSEHGNNAFFYYFEHRSSKLPWPEWMGVMHG  439
hBCHE-3   330  LVYGAPGFSKDNNSIITRKEFQEGLKIFFPGVSEFGKESILFHYTDWVDEQRPENYREALGDVVGDYFFICPALEFAKKFSEHGNNAFFYYFEHRSSKLPWPEWMGVMHG  439
hBCHE-4   330  LVYGAPGFSKDNNSIITRKEFQEGLKIFFPNVSEFGKESILFHYTDWVDEQRPENYREALGDVVGDYFFICPALEFAKKFSEHGNNAFFYYFEHRSSKLPWPEWMGVMHG  439
hBCHE-5   330  LVYGAPGFSKDNNSIITRKEFQEGLKIFFPNVSEFGKESILFHYTDWVDEQRPENYREALGDVVGDYFFICPALEFAKKYSEHGNNAFFYYFEHRSSKLPWPEWMGVMHG  439
hBCHE-6   330  LVYGAPGFSKDNNSIITRKEFQEGLKVFFPNVSEFGKESILFHYTDWEDEDRPENYREALGEVVGDYFFICPALEFAKKYSEHGNNAYFYYFEHRSSKLPWPEWMGVMHG  439
hBCHE-7   330  LVYGAPGFSKDNDSIITRKEFQEGLKVFFPNVSEFGKESILFHYTDWEDEDRPENYRDALAEVVGDYFFICPALEFAKKYAEHGNNAYFYYFEHRSSKLPWPEWMGVMHG  439

hBCHE-WT  440  YEIEFVFGLPLERRDNYTKAEEILSRSIVKRWANFAKYGNPNETQNNSTSWPVFKSTEQKYLTLNTESTRIMTKLRAQQCRFWTSFFPKV                      529
hBCHE-1   440  YEIEFVFGLPLERRLNYTKAEEILSRKIMKRWANFAKYGNPNETQNNSTSWPVFKSTEQKYLTLNTESTRIMTKLRAQQCRFWTSFFPKV                      529
hBCHE-2   440  YEIEFVFGLPLERRKNYTKAEEILSREIMKRWANFAKYGNPNETQNNSTSWPVFKSTEQKYLTLNTESTRIMTKLRAQQCRFWTSFFPKV                      529
hBCHE-3   440  YEIEFVFGLPLERRLNYTKAEEILSREIMKRWANFAKYGNPNETQNNSTSWPVFKSTEQKYLTLNTESTRIMTKLRAQQCRFWTSFFPKV                      529
hBCHE-4   440  YEIEFVFGLPLERRLNYTKAEEILSREIMRRWANFAKYGNPNETQNNSTSWPVFKSTEQKYLTLNTESTRIMTKLRAQQCRFWTSFFPKV                      529
hBCHE-5   440  YEIEFVFGLPLERRLNYTKAEEILSREIMRRWANFAKYGNPNETQNNSTQWPVFKSTEQKYLTLNTESTRIMTKLRAQQCRFWKSFFPKV                      529
hBCHE-6   440  YEIEFVFGLPLERRLNYTKAEEILSREIMRRWANFAKYGNPNETQNNSTQWPVFKSTEQKYLTLNTESTRIMTKLRAQHCRFWKSFFPKV                      529
hBCHE-7   440  YEIEFVFGLPLERRLNYTKEEEILSREIMRRWANFAKYGNPNETQNNSTQWPVFKPTEQKYLTLNTESSRIMTKLRAQHCRFWNSFFPKV                      529
```

Figure S2. Bacterial optimized sequences of the 7 constructs proposed by the *PROSS* process. In bold and underlined are the respective KpnI and XhoI restriction sites for pThioHis vector cloning.

hBChE-1

GGTACCCATGGAAGATGACATTATCATCACCACCAAAAATGGTAAAGTGCGTGGTATGAATCTGACCGTGTTTGG
TGGCACCGTTACCGCATTTCTGGGTATTCCGTATGCACAGCCTCCGCTGGGTCGTCTGCGTTTCAAAAAACCGCA
GCCGCTGACCAAATGGTCAGGTATTTGGAATGCAACCAAATATGCAAATAGCTGCTGCCAGAATATTGATCAGAG
CTTTCCGGGTTTTCATGGTAGCGAAATGTGGAATCCGAATACCGATCTGAGCGAAGATTGTCTGTATCTGAATGT
TTGGATTCCGGCACCGAAACCGAAAAATGCAACCGTTCTGATTTGGATTTATGGCGGTGGTTTTCAGACCGGCAC
CAGCAGCCTGCATGTTTATGATGGTAAATTTCTGGCACGTGTGGAACGTGTTATTGTTGTGAGCATGAATTATCG
TGTTGGTGCACTGGGTTTTCTGGCCCTGCCTGGTAATCCGGAAGCACCGGGTAATATGGGTCTGTTTGATCAGCA
GCTGGCGCTGCAGTGGGTTCAGAAAAACATTGCAGCATTTGGCGGTAATCCGAAAAGCGTTACCCTGTTTGGTGA
AAGTGCCGGTGCAGCAAGCGTTAGCCTGCATCTGCTGAGTCCGGGTAGCCATCCGCTGTTTACCCGTGCAATTCT
GCAGAGCGGTAGCTTTAATGCACCGTGGGCAGTTACCAGTCTGTATGAAGCACGTAATCGTACCCTGAATCTGGC
AAAACTGCTGGGTTGTAGCCGTGAAAATGAAACCGAGATTATCAAATGCCTGCGCAATAAAGATCCGCAAGAAAT
TCTGCTGAATGAAGCATTTGTTGTGCCGTATGGTACACCGCTGAGCGTTAATTTTGGTCCGACCGTTGATGGTGA
TTTTCTGACCGATATGCCGGATATTCTGCTGGAACTGGGTCAGTTCAAAAAAACCCAGATTCTGGTTGGCGTGAA
TAAAGATGAAGGCACCGCCTTTCTGGTTTATGGCGCACCGGGTTTTAGCAAAGATAACAATAGCATTATCACCCG
CAAAGAATTTCAAGAGGGCCTGAAAATCTTTTTTCCGGGTGTTAGCGAATTTGGCAAAGAAAGCATTCTGTTCCA
TTATACCGATTGGGTTGATGATCAGCGTCCGGAAAACTATCGTGAAGCACTGGGTGATGTTGTTGGCGATTATTT
CTTTATTTGTCCGGCACTGGAATTTACCAAAAAATTCAGCGAATGGGGCAACAACGCCTTTTTCTATTATTTCGA
ACATCGGAGCAGCAAACTGCCGTGGCCTGAATGGATGGGTGTTATGCATGGTTATGAAATCGAATTTGTTTTTGG
TCTGCCGCTGGAACGTCGTCTGAACTATACCAAAGCCGAAGAAATCCTGAGCCGTAAGATTATGAAACGTTGGGC
AAATTTTGCGAAATATGGCAACCCGAATGAAACCCAGAATAATAGCACCAGCTGGCCTGTGTTTAAAAGCACCGA
ACAGAAATATCTGACCCTGAATACCGAAAGCACCCGTATTATGACCAAACTGCGTGCCCAGCAGTGTCGTTTTTG
GACCAGTTTTTTTCCGAAAGTGTAATAACTCGAG

hBChE-2

GGTACCCATGGAAGATGACATTATCATCACCACCAAAAATGGTAAAGTGCGTGGTATGAATCTGACCGTGTTTGG
TGGCACCGTTACCGCATTTCTGGGTATTCCGTATGCACAGCCTCCGCTGGGTCGTCTGCGTTTCAAAAAACCGCA
GCCGCTGACCAAATGGTCAGGTATTTGGAATGCAACCAAATATCCGAATAGCTGCTGCCAGAATATTGATCAGAG
CTTTCCGGGTTTTCATGGTAGCGAAATGTGGAATCCGAATACCGATCTGAGCGAAGATTGTCTGTATCTGAATGT
TTGGATTCCGGCACCGAAACCGAAAAATGCAACCGTTATGATTTGGATTTATGGCGGTGGTTTTCAGACCGGCAC
CAGCAGCCTGCATGTTTATGATGGTAAATTTCTGGCACGTGTGGAACGTGTTATTGTTGTGAGCATGAATTATCG
TGTTGGTGCACTGGGTTTTCTGGCCCTGCCTGGTAATCCGGAAGCACCGGGTAATATGGGTCTGTTTGATCAGCA
GCTGGCGCTGCAGTGGGTTCAGAAAAACATTGCAGCATTTGGCGGTAATCCGAAAAGCGTTACCCTGTTTGGTGA
AAGTGCCGGTGCAGCAAGCGTTAGCCTGCATCTGCTGAGTCCGGGTAGCCATCCGCTGTTTACCCGTGCAATTCT
GCAGAGCGGTAGCTTTAATGCACCGTGGGCAGTTACCAGTCCGGAAGAAGCACGTAATCGTACCCTGAATCTGGC
AAAACTGCTGGGTTGTAGCCGTGAAAATGAAACCGAGATTATCAAATGCCTGCGCAATAAAGATCCGCAAGAAAT
TCTGCTGAATGAAGCATTTGTTGTGCCGTATGGTACACCGCTGAGCGTTAATTTTGGTCCGACCGTTGATGGTGA
TTTTCTGACCGATATGCCGGATATTCTGCTGGAACTGGGTCAGTTCAAAAAAACCCAGATTCTGGTTGGCGTGAA
TAAAGATGAAGGCACCGCCTTTCTGGTTTATGGCGCACCGGGTTTTAGCAAAGATAACAATAGCATTATCACCCG
CAAAGAATTTCAAGAGGGCCTGAAAATCTTTTTTCCGGGTGTTAGCGAATTTGGCAAAGAAAGCATTCTGTTCCA
TTATACCGATTGGGTTGATGATCAGCGTCCGGAAAACTATCGTGAAGCACTGGGTGATGTTGTTGGCGATTATTT
CTTTATTTGTCCGGCACTGGAATTTGCCAAAAAATTCAGCGAACATGGCAACAACGCCTTTTTCTATTATTTCGA
ACATCGGAGCAGCAAACTGCCGTGGCCTGAATGGATGGGTGTTATGCATGGTTATGAAATCGAATTTGTTTTTGG
TCTGCCGCTGGAACGTCGTAAAAACTATACCAAAGCCGAAGAAATCCTGAGCCGTGAGATTATGAAACGTTGGGC
AAATTTTGCGAAATATGGCAACCCGAATGAAACCCAGAATAATAGCACCAGCTGGCCTGTGTTTAAAAGCACCGA
ACAGAAATATCTGACCCTGAATACCGAAAGCACCCGTATTATGACCAAACTGCGTGCCCAGCAGTGTCGTTTTTG
GACCAGTTTTTTTCCGAAAGTGTAATAACTCGAG

hBChE-3

GGTACCCATGGAAGATGACATTATCATCACCACCAAAAATGGTAAAGTGCGTGGTATGAATCTGACCGTGTTTGG
TGGCACCGTTACCGCATTTCTGGGTATTCCGTATGCACAGCCTCCGCTGGGTCGTCTGCGTTTCAAAAAACCGCA
GCCGCTGACCAAATGGTCAGGTATTTGGAATGCAACCAAATATGCCAATAGCTGCTGCCAGAATATTGATCAGAG
CTTTCCGGGTTTTCATGGTAGCGAAATGTGGAATCCGAATACCGATCTGAGCGAAGATTGTCTGTATCTGAATGT
TTGGATTCCGGCACCGAAACCGAAAAATGCAACCGTTATGATTTGGATTTATGGCGGTGGTTTTCAGACCGGCAC
CAGCAGCCTGCCGGTTTATGATGGTAAATTTCTGGCACGTGTTGAACGTGTTATTGTGGTGAGCATGAATTATCG

TGTTGGTGCACTGGGTTTTCTGGCCCTGCCTGGTAATCCGGAAGCACCGGGTAATATGGGTCTGTTTGATCAGCA
GCTGGCGCTGCAGTGGGTTCAAGATAACATTGCAGCATTTGGCGGTAATCCGAAAAGCGTTACCCTGTTTGGTGA
AAGTGCCGGTGCAGCAAGCGTTAGCCTGCATCTGCTGAGTCCGGGTAGCCATCCGCTGTTTACCCGTGCAATTCT
GCAGAGCGGTAGCTTTAATGCACCGTGGGCAGTTACCAGTCCGGAAGAAGCACGTAATCGTACCCTGAATCTGGC
AAAACTGCTGGGTTGTAGCCGTGAAAATGAAACCGAGATTATCAAATGCCTGCGCAATAAAGATCCGCAAGAAAT
TCTGGATAATGAAGCCTTTGTTGTGCCGTATGGTACACCGCTGAGCGTTAATTTTGGTCCGACCGTTGATGGTGA
TTTTCTGACCGATATGCCGGATATTCTGCTGGAACTGGGTCAGTTCAAAAAAACCCAGATTCTGGTTGGCGTGAA
TAAAGATGAAGGCACCGCCTTTCTGGTTTATGGCGCACCGGGTTTTAGCAAAGATAACAATAGCATTATCACCCG
CAAAGAATTTCAAGAGGGCCTGAAAATCTTTTTTCCGGGTGTTAGCGAATTTGGCAAAGAAAGCATCCTGTTTCA
TTATACCGATTGGGTTGATGAACAGCGTCCGGAAAACTATCGTGAAGCACTGGGTGATGTTGTTGGCGATTATTT
CTTTATTTGTCCGGCACTGGAATTTGCCAAAAAATTCAGCGAACATGGCAACAACGCCTTTTTCTATTATTTCGA
ACATCGGAGCAGCAAACTGCCGTGGCCTGAATGGATGGGTGTTATGCATGGTTATGAAATCGAATTTGTTTTTGG
TCTGCCGCTGGAACGTCGTCTGAATTATACCAAAGCCGAAGAAATCCTGAGTCGCGAAATTATGAAACGTTGGGC
AAATTTTGCGAAATATGGTAACCCGAATGAAACCCAGAATAATAGCACCAGCTGGCCTGTGTTTAAAAGCACCGA
ACAGAAATATCTGACCCTGAATACCGAAAGCACCCGTATTATGACCAAACTGCGTGCCCAGCAGTGTCGTTTTTG
GACCAGCTTTTTTCCCAAAGTGTGATAACTCGAG

hBChE-4

GGTACCCATGGAAGATGACATTATCATCACCACCAAAAATGGTAAAGTGCGTGGTATGAATCTGACCGTGTTTGG
TGGCACCGTTACCGCATTTCTGGGTATTCCGTATGCACAGCCTCCGCTGGGTCGTCTGCGTTTCAAAAAACCGCA
GCCGCTGACCAAATGGTCAGGTATTTGGAATGCAACCAAATATGCCAATAGCTGCTGCCAGAATATTGATCAGAG
CTTTCCGGGTTTTCATGGTAGCGAAATGTGGAATCCGAATACCGATCTGAGCGAAGATTGTCTGTATCTGAATGT
TTGGATTCCGGCACCGAAACCGAAAAATGCAACCGTTATGATTTGGATTTATGGCGGTGGTTTTCAGACCGGCAC
CAGCAGCCTGCCGGTTTATGATGGTAAATTTCTGGCACGTGTTGAACGTGTTATTGTGGTGAGCATGAATTATCG
TGTTGGTGCACTGGGTTTTCTGGCCCTGCCTGGTAATCCGGAAGCACCGGGTAATATGGGTCTGTTTGATCAGCA
GCTGGCGCTGCAGTGGGTTCAAGATAACATTGCAGCATTTGGCGGTAATCCGAAAAGCGTTACCCTGTTTGGTGA
AAGTGCCGGTGCAGCAAGCGTTAGCCTGCATCTGCTGAGTCCGGGTAGCCATCCGCTGTTTACCCGTGCAATTCT
GCAGAGCGGTAGCGCAAATGCACCGTGGGCAGTTACCAGTCCGGAAGAAGCACGTAATCGTACCCTGAATCTGGC
AAAACTGCTGGGTTGTAGCCGTGAAAATGAAACCGAGATTATCAAATGCCTGCGCAATAAAGATCCGCAAGAAAT
TCTGGATAATGAAGCCTTTGTTGTGCCGTATGGTACACCGCTGAGCGTTAATTTTGGTCCGACCGTTGATGGTGA
TTTTCTGACCGATATGCCGGATATTCTGCTGGAACTGGGTCAGTTCAAAAAAACCCAGATTCTGGTTGGCGTGAA
TAAAGATGAAGGCACCGCCTTTCTGGTTTATGGCGCACCGGGTTTTAGCAAAGATAACAATAGCATTATCACCCG
CAAAGAATTTCAAGAGGGCCTGAAAATCTTTTTTCCGAACGTTAGCGAATTTGGCAAAGAAAGCATCCTGTTTCA
TTATACCGATTGGGTTGATGAACAGCGTCCGGAAAACTATCGTGAAGCACTGGGTGATGTTGTTGGCGATTATTT
CTTTATTTGTCCGGCACTGGAATTTGCCAAAAAATTCAGCGAACATGGCAACAACGCCTTTTTCTATTATTTCGA
ACATCGGAGCAGCAAACTGCCGTGGCCTGAATGGATGGGTGTTATGCATGGTTATGAAATCGAATTTGTTTTTGG
TCTGCCGCTGGAACGTCGTCTGAATTATACCAAAGCCGAAGAAATCCTGAGTCGCGAAATTATGCGTCGTTGGGC
AAATTTTGCGAAATATGGTAACCCGAATGAAACCCAGAATAATAGCACCAGCTGGCCTGTGTTTAAAAGCACCGA
ACAGAAATATCTGACCCTGAATACCGAAAGCACCCGTATTATGACCAAACTGCGTGCCCAGCAGTGTCGTTTTTG
GACCAGCTTTTTTCCCAAAGTGTGATAACTCGAG

hBChE-5

GGTACCCATGGAAGATGACATTATCATCACCACCAAAAATGGTAAAGTGCGTGGTATGAATCTGACCGTGTTTGG
TGGCACCGTTACCGCATTTCTGGGTATTCCGTATGCACAGCCTCCGCTGGGTCGTCTGCGTTTCAAAAAACCGCA
GCCGCTGACCAAATGGTCAGGTATTTGGAATGCAACCAAATATGCCAATAGCTGCTGCCAGAATATTGATCAGAG
CTTTCCGGGTTTTCATGGTAGCGAAATGTGGAATCCGAATACCGATCTGAGCGAAGATTGTCTGTATCTGAATGT
TTGGATTCCGGCACCGAAACCGAAAAATGCAACCGTTATGGTTTGGATTTATGGCGGTGGTTTTCAGACCGGCAC
CAGCAGCCTGCCGGTTTATGATGGTAAATTTCTGGCACGTGTTGAACGTGTTATTGTGGTGAGCATGAATTATCG
TGTTGGTGCACTGGGTTTTCTGGCCCTGCCTGGTAATCCGGAAGCACCGGGTAATATGGGTCTGTTTGATCAGCA
GCTGGCGCTGCAGTGGGTTCAAGATAACATTGCAGCATTTGGCGGTAATCCGAAAAGCGTTACCCTGTTTGGTGA
AAGTGCCGGTGCAGCAAGCGTTAGCCTGCATCTGCTGAGTCCGGGTAGCCATCCGCTGTTTACCCGTGCAATTCT
GCAGAGCGGTAGCGCAAATGCACCGTGGGCAGTTACCAGTCCGGAAGAAGCACGTAATCGTACCCTGAATCTGGC
AAAACTGCTGGGTTGTAGCCGTGAAAATGAAACCGAGATTATCAAATGCCTGCGCAATAAAGATCCGCAAGAAAT
TCTGGATAATGAAGCCTTTGTTGTGCCGTATGGTACACCGCTGAGCGTTAATTTTGGTCCGACCGTTGATGGTGA
TTTTCTGACCGATATGCCGGATATTCTGCTGGAACTGGGTCAGTTCAAAAAAACCCAGATTCTGGTTGGCGTGAA
TAAAGATGAAGGCACCGCCTTTCTGGTTTATGGCGCACCGGGTTTTAGCAAAGATAACAATAGCATTATCACCCG
CAAAGAATTTCAAGAGGGCCTGAAAATCTTTTTTCCGAACGTTAGCGAATTTGGCAAAGAAAGCATCCTGTTTCA
TTATACCGATTGGGTTGATGAACAGCGTCCGGAAAACTATCGTGAAGCACTGGGTGATGTTGTTGGCGATTATTT
CTTTATTTGTCCGGCACTGGAATTTGCCAAAAAATACAGCGAACATGGCAACAACGCCTTTTTCTATTATTTCGA
ACATCGGAGCAGCAAACTGCCGTGGCCTGAATGGATGGGTGTTATGCATGGTTATGAAATCGAATTTGTTTTTGG

TCTGCCGCTGGAACGTCGTCTGAATTATACCAAAGCCGAAGAAATCCTGAGTCGCGAAATTATGCGTCGTTGGGC
AAATTTTGCGAAATATGGTAACCCGAATGAAACCCAGAATAATAGCACCCAGTGGCCTGTGTTTAAAAGCACCGA
ACAGAAATATCTGACCCTGAATACCGAAAGCACCCGTATTATGACCAAACTGCGTGCCCAGCAGTGTCGTTTTTG
GAAAAGCTTTTTTCCCAAAGTGTGATAACTCGAG

hBChE-6

GGTACCCATGGAAGATGACATTATCATCACCACCAAAAATGGTAAAGTGCGTGGTATGAATCTGACCGTGTTTGG
TGGCACCGTTACCGCATTTCTGGGTATTCCGTATGCACAGCCTCCGCTGGGTCGTCTGCGTTTCAAAAAACCGCA
GCCGCTGACCAAATGGTCAGGTATTTGGAATGCAACCAAATATGCCAATAGCTGTATGCAGAATATCGATACCAG
CTTTCCGGGTTTTCATGGTAGCGAAATGTGGAATCCGAATACCGATCTGAGCGAAGATTGTCTGTATCTGAATGT
TTGGATTCCGGCACCGAAACCGAAAAATGCAACCGTTATGGTTTGGATTTATGGTGGTGGTTTTCAGACCGGCAC
CAGCAGCCTGCCGGTTTATGATGGTAAATTTCTGGCACGTGTTGAACGTGTTATTGTGGTGAGCATGAATTATCG
TGTTGGTGCACTGGGTTTTCTGGCCCTGCCTGGTAATCCGGAAGCACCGGGTAATATGGGTCTGTTTGATCAGCA
GCTGGCACTGAAATGGGTTCAAGATAACATTGCAGCATTTGGCGGTAATCCGAAAAGCGTTACCCTGTTTGGTGA
AAGTGCCGGTGCAGCAAGCGTTAGCCTGCATCTGCTGAGTCCGGGTAGCCATCCGCTGTTTACCCGTGCAATTCT
GCAGAGCGGTAGCGCAAATGCACCGTGGGCAGTTATGAGTCCGGAAGAAGCACGTAATCGTACCCTGAATCTGGC
AAAACTGCTGGGTTGTAGCCGTGAAAATGAAACCGAGATTATCAAATGCCTGCGCAATAAAGATCCGCAAGAAAT
TCTGGATAATGAAGCCTTTGTTGTGCCGTATGGTACACCGCTGAGCGTTAATTTTGGTCCGACCGTTGATGGTGA
TTTTCTGACCGATATGCCGGATACACTGCTGGAACTGGGTCAGTTCAAAAAAACCCAGATTCTGGTTGGCGTGAA
TAAAGATGAAGGCACCGCCTTTCTGGTTTATGGCGCACCGGGTTTTAGCAAAGATAACAATAGCATTATCACCCG
CAAAGAATTTCAAGAGGGTCTGAAAGTGTTTTTTCCGAACGTTAGCGAATTTGGCAAAGAAAGCATCCTGTTTCA
TTATACCGATTGGGAAGATGAAGATCGTCCGGAAAACTATCGTGAAGCACTGGGTGAAGTTGTTGGCGATTATTT
CTTTATTTGTCCGGCACTGGAATTTGCCAAAAAATACAGCGAACATGGCAACAACGCCTACTTCTATTATTTCGA
ACATCGTAGCAGCAAACTGCCGTGGCCTGAATGGATGGGTGTTATGCATGGTTATGAAATCGAATTTGTTTTTGG
TCTGCCGCTGGAACGTCGTCTGAATTATACCAAAGCCGAAGAAATCCTGAGTCGCGAAATTATGCGTCGTTGGGC
AAATTTTGCGAAATATGGTAACCCGAATGAAACCCAGAATAATAGCACCCAGTGGCCTGTGTTTAAAAGCACCGA
ACAGAAATATCTGACCCTGAATACCGAAAGCACCCGTATTATGACCAAACTGCGTGCACAGCATTGTCGTTTTTG
GAAAAGCTTCTTTCCGAAGGTGTGATAACTCGAG

hBChE-7

GGTACCCATGGAAGATGACATTATCATCACCACCAAAAATGGTAAAGTGCGTGGTATGAATCTGACCGTGTTTGG
TGGCACCGTTACCGCATTTCTGGGTATTCCGTATGCACAGCCTCCGCTGGGTCGTCTGCGTTTCAAAAAACCGCA
GCCGCTGACCAAATGGTCAGGTATTTGGAATGCAACCAAATATGCCAATAGCTGTATGCAGAATATCGATACCAG
CTTTCCGGGTTTTCATGGTAGCGAAATGTGGAATCCGAATACCGATCTGAGCGAAGATTGTCTGTATCTGAATGT
TTGGATTCCGGCACCGAAACCGAAAAATGCAACCGTTATGGTTTGGATTTATGGTGGTGGTTTTCAGACCGGCAC
CAGCAGCCTGCCGGTTTATGATGGTAAATTTCTGGCACGTGTTGAACGTGTTATTGTGGTGAGCATGAATTATCG
TGTTGGTGCACTGGGTTTTCTGGCCCTGCCTGGTAATCCGGAAGCACCGGGTAATATGGGTCTGTTTGATCAGCA
GCTGGCACTGAAATGGGTTCAAGATAACATTGCAGCATTTGGTGGCGATCCGAATCGTGTTACCCTGTTTGGTGA
AAGTGCCGGTGCAGCAAGCGTTAGCCTGCATCTGCTGAGTCCGGGTAGCCATCCGCTGTTTACCCGTGCAATTCT
GCAGAGCGGTAGCGCAAATGCACCGTGGGCAGTTATGAGTCCGGAAGAAGCACGTAATCGTACCCTGAATCTGGC
AAAACTGCTGGGTTGTAGCCGTGAAAATGAAACCGAGATTATCAAATGCCTGCGCAATAAAGATCCGCAAGAAAT
TCTGGATAATGAAGCCTTTGTTGTGCCGTATAGCACACCGCTGAGCGTTAATTTTGGTCCGACCGTTGATGGTGA
TTTTCTGACCGATATGCCGGATACACTGCTGGAACTGGGTCAGTTCAAAAAAACCCAGATTCTGGTTGGCGTGAA
TAAAGATGAAGGCACCGCCTTTCTGGTTTATGGCGCACCGGGTTTTAGCAAAGATAACGATAGCATTATTACCCG
CAAAGAATTTCAAGAGGGCCTGAAAGTTTTTTTTCCGAACGTTAGCGAATTTGGCAAAGAAAGCATCCTGTTTCA
TTATACCGATTGGGAAGATGAAGATCGTCCGGAAAACTATCGTGATGCACTGGCCGAAGTTGTTGGTGATTATTT
CTTTATTTGTCCGGCACTGGAATTTGCCAAAAAATACGCAGAACATGGCAACAACGCCTACTTCTATTATTTCGA
ACATCGTAGCAGCAAACTGCCGTGGCCTGAATGGATGGGTGTTATGCATGGTTATGAAATCGAATTTGTTTTTGG
TCTGCCGCTGGAACGTCGTCTGAATTATACCAAAGAAGAAGAAATCCTGAGTCGCGAAATTATGCGTCGTTGGGC
AAATTTTGCGAAATATGGTAACCCGAATGAAACCCAGAATAATAGCACCCAGTGGCCTGTTTTTAAACCGACCGA
ACAGAAATATCTGACCCTGAATACCGAAAGCAGCCGTATTATGACCAAACTGCGTGCACAGCATTGTCGTTTTTG
GAATAGCTTTTTCCCGAAGGTGTGATAACTCGAG

Figure S3. Determination of kinetic parameters of hBChE-7 for BTC hydrolysis. Upper panel, enzymatic activity titration by increasing concentration of substrate. Lower panel, titration of residual activity by increasing concentration of echothiophate.

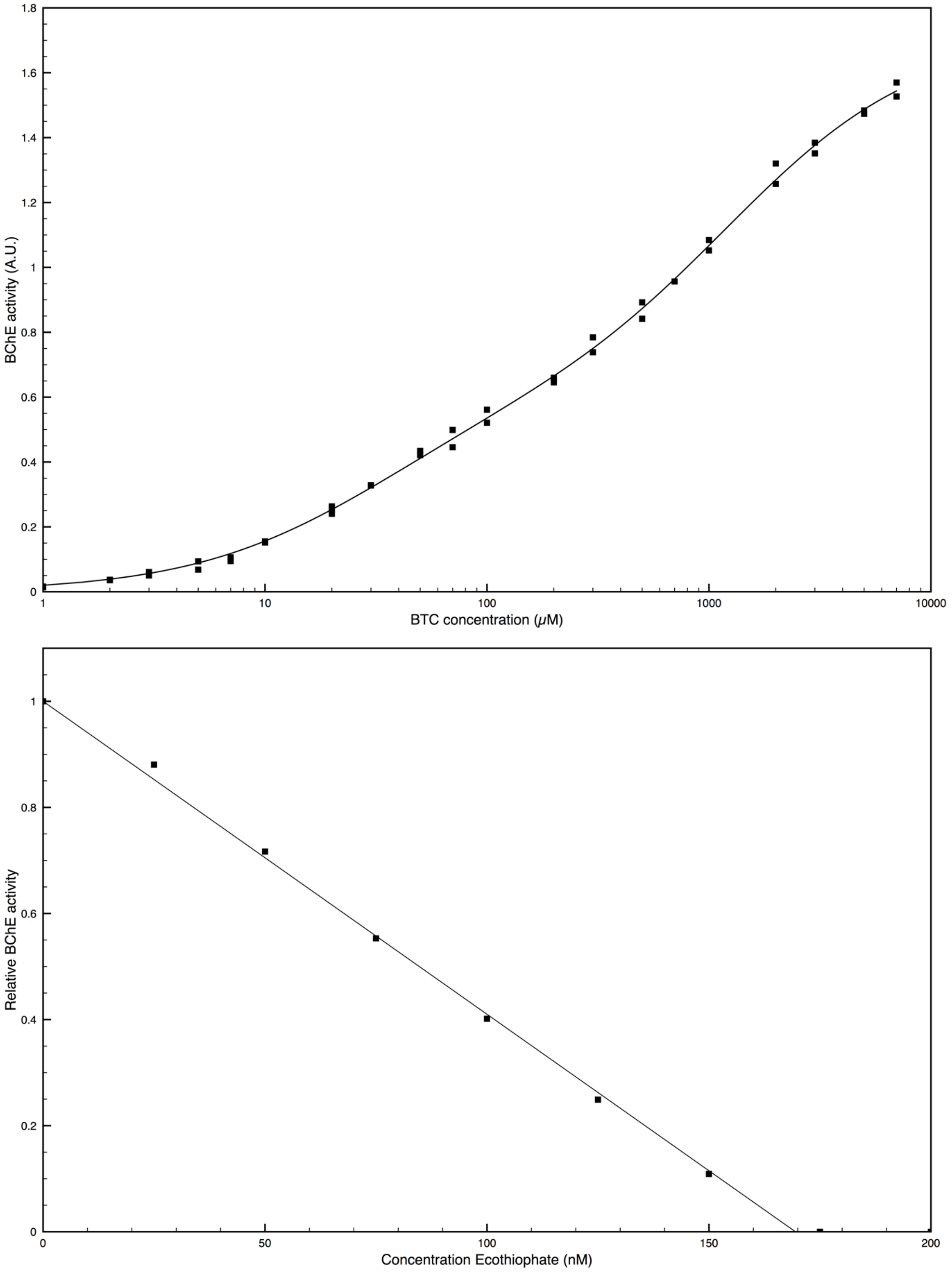

Figure S4. Effects of different buffers on hBChE-7 thermal stability. Examples of normalized DSF scans of hBChE-7 in different buffer conditions, without (plain trace) or with 0.25M NaCl (dashed trace). 0.1M KH_2PO_4 pH 6.0, red trace; 0.1M Cacodylate pH 6.5, blue trace; 0.1M NaH_2PO_4 pH 7.5, green trace; 0.1M HEPES pH 7.5, magenta trace. For HEPES, increasing concentrations of NaCl, show the stabilizing effect of the buffer ionic strength.

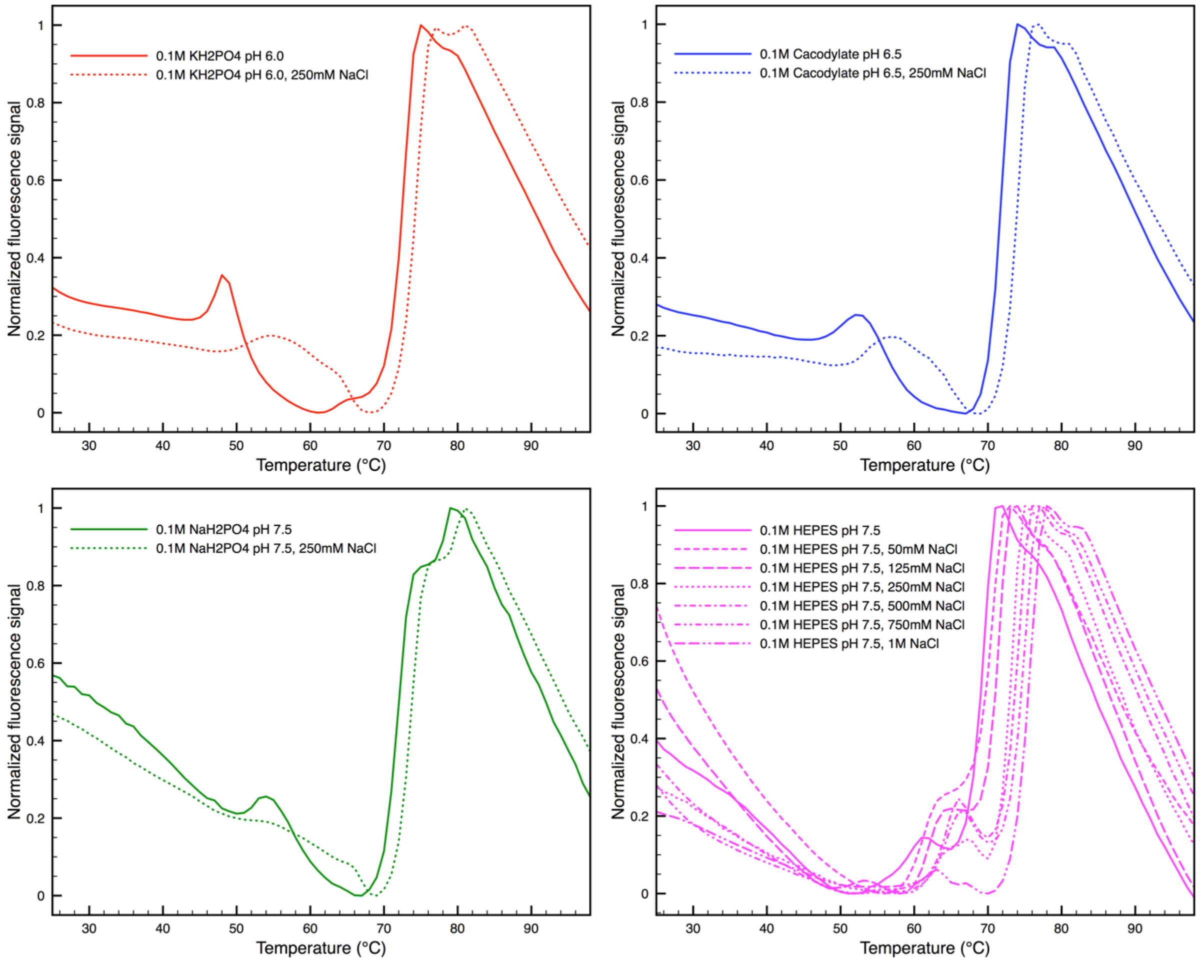

Figure S5. Composition of the buffer screen used in the DSF assay

	1	2	3	4	5	6	7	8	9	10	11	12
**A**	Water	Citrate pH 4.0	Na Acetate pH 4.5	Citrate pH 5.0	MES pH 6.0	KH2PO4 pH 6.0	Citrate pH 6.0	Bis-Tris pH 6.5	Cacodylate pH 6.5	NaH2PO4 pH 7.0	KH2PO4 pH 7.0	HEPES pH 7.0
**B**	MOPS pH 7.0	Am. Acetate pH 7.3	Tris-HCl pH 7.5	NaH2PO4 pH 7.5	HEPES pH 7.5	Imidazole pH 8.0	Tris-HCl pH 8.0	Tricine pH 8.0	Bicine pH 8.0	Bicine pH 8.5	Tris-HCl pH 8.5	Bicine pH 9.0
**C**	Water 250mM NaCl	Citrate pH 4.0 250mM NaCl	Na Acetate pH 4.5 250mM NaCl	Citrate pH 5.0 250mM NaCl	MES pH 6.0 250mM NaCl	KH2PO4 pH 6.0 250mM NaCl	Citrate pH 6.0 250mM NaCl	Bis-Tris pH 6.5 250mM NaCl	Cacodylate pH 6.5 250mM NaCl	NaH2PO4 pH 7.0 250mM NaCl	KH2PO4 pH 7.0 250mM NaCl	HEPES pH 7.0 250mM NaCl
**D**	MOPS pH 7.0 250mM NaCl	Am. Acetate pH 7.3 250mM NaCl	Tris-HCl pH 7.5 250mM NaCl	NaH2PO4 pH 7.5 250mM NaCl	HEPES pH 7.5 250mM NaCl	Imidazole pH 8.0 250mM NaCl	Tris-HCl pH 8.0 250mM NaCl	Tricine pH 8.0 250mM NaCl	Bicine pH 8.0 250mM NaCl	Bicine pH 8.5 250mM NaCl	Tris-HCl pH 8.5 250mM NaCl	Bicine pH 9.0 250mM NaCl
**E**	10mM MES pH 6.0	50mM MES pH 6.0	100mM MES pH 6.0	250mM MES pH 6.0	10mM KH2PO4 pH 6.0	50 mM KH2PO4 pH 6.0	100m KH2PO4 pH 6.0	250mM KH2PO4 pH 6.0	10mM Citrate pH 6.0	50 mM Citrate pH 6.0	100mM Citrate pH 6.0	250mM Citrate pH 6.0
**F**	10mM NaH2PO4 pH 7.5	50mM NaH2PO4 pH 7.5	100mM NaH2PO4 pH 7.5	250mM NaH2PO4 pH 7.5	10mM HEPES pH 7.5	50mM HEPES pH 7.5	100mM HEPES pH 7.5	250mM HEPES pH 7.5	10mM Tris-HCl pH 7.5	50mM Tris-HCl pH 7.5	100mM Tris-HCl pH 7.5	250mM Tris-HCl pH 7.5
**G**	50mM MES pH 6.0 50mM NaCl	50mM MES pH 6.0 125mM NaCl	50mM MES pH 6.0 250mM NaCl	50mM MES pH 6.0 500mM NaCl	50mM MES pH 6.0 750mM NaCl	50mM MES pH 6.0 1M NaCl	50mM MOPS pH 7.0 50mM NaCl	50mM MOPS pH 7.0 125mM NaCl	50mM MOPS pH 7.0 250mM NaCl	50mM MOPS pH 7.0 500mM NaCl	50mM MOPS pH 7.0 750mM NaCl	50mM MOPS pH 7.0 1M NaCl
**H**	50mM HEPES pH 7.5 50mM NaCl	50mM HEPES pH 7.5 125mM NaCl	50mM HEPES pH 7.5 250mM NaCl	50mM HEPES pH 7.5 500mM NaCl	50mM HEPES pH 7.5 750mM NaCl	50mM HEPES pH 7.5 1M NaCl	50mM Tris-HCl pH 8.0 50mM NaCl	50mM Tris-HCl pH 8.0 125mM NaCl	50mM Tris-HCl pH 8.0 250mM NaCl	50mM Tris-HCl pH 8.0 500mM NaCl	50mM Tris-HCl pH 8.0 750mM NaCl	50mM Tris-HCl pH 8.0 1M NaCl
